# Clinical Characteristics and Prognosis of Anti-AChR Positive Myasthenia Gravis Combined With Anti-LRP4 or Anti-Titin Antibody

**DOI:** 10.3389/fneur.2022.873599

**Published:** 2022-05-09

**Authors:** Yuping Chen, Xiaoyong Tao, Yan Wang, Shengjie Xu, Yanhua Yang, Jinming Han, Feng Qiu

**Affiliations:** ^1^Senior Department of Neurology, The First Medical Center of PLA General Hospital, Beijing, China; ^2^Department of Neurology, Xuanwu Hospital, Capital Medical University, Beijing, China

**Keywords:** acetylcholine receptor, Myasthenia Gravis, low-density lipoprotein receptor-related protein 4, Titin antibody, minimal manifestations status (MMS)

## Abstract

**Objective:**

This study aimed to summarize the clinical characteristics and prognosis of patients with anti- acetylcholine receptor (AChR) positive myasthenia gravis (MG) with a combination of anti-LRP4 or Titin antibodies.

**Methods:**

A total of 188 patients with generalized MG before immunotherapy were retrospectively collected and then divided into three groups: single anti-AChR positive-MG (AChR-MG, 101 cases), anti-AChR combined with anti-low-density lipoprotein receptor-related protein four-positive MG (AChR+LRP4-MG, 29 cases), and anti-AChR combined with anti-Titin-positive MG (AChR+Titin-MG, 58 cases). Clinical manifestations, therapeutic responses to immunotherapy, and follow-up information were analyzed.

**Results:**

Of the 188 seropositive MG patients, 29 (15.4%) were positive for both AChR and LRP4 antibodies, and 58 (30.9%) were positive for both AChR and Titin antibodies. The mean disease onset ages in the three groups were 47.41 ± 7.0, 49.81 ± 9.2, and 48.11 ± 6.5 years, respectively. AChR+LRP4-MG showed female predominance (27.6% were males and 72.4% were females), with mild overall clinical symptoms. The AChR+Titin-MG group showed shorter times for conversion to generalized MG (5.14 ± 0.0 months) than the AChR-MG group (11.69 ± 0.0 months) and the AChR+LRP4-MG group (13.08 ± 0.5 months; *P* < 0.001 in both cases). Furthermore, AChR+Titin-MG group had increased bulbar dysfunction, higher incidences of thymoma (32.8 vs. 19.8% and 3.4%, *P*=0.035), more severe quantitative MG scores, as assessed by both QMG scores [15.5 (11.75–22.5) vs. 13 (8–19), *P* = 0.005; and 9 (6–14) *P* < 0.001], and MG-ADL scores [10 (8–13) vs. 8 (5–13), *P* = 0.018; and 6 (4–8), *P* < 0.001]. Treatment for AChR+Titin-MG was largely dependent on corticosteroids and immunosuppressive agents (56.7 vs. 19.2% and 16.7%, *p* = 0.028). The rates of achieving s(MMS) or better within 2 years following immunotherapy in the three groups were 51.5, 62.1, and 51.7%, respectively (*P* = 0.581).

**Conclusion:**

Clinical symptoms of anti-AChR positive MG combined with Titin antibody were more severe and progressed faster than those in the AChR + LRP4 and AChR groups. Regardless of antibody status, all patients responded well to immunotherapy and had relatively good prognoses.

## Introduction

Myasthenia gravis (MG) is an autoimmune disease involving antibody-mediated destruction of the neuromuscular junction, which causes fatigable weakness (1). There are three confirmed pathogenic antibodies in MG: acetylcholine receptor antibody (AChR-Ab), muscle-specific tyrosine kinase antibody (MuSK-Ab), and low-density lipoprotein receptor-related protein 4 antibody (LRP4-Ab) (1–3). These main concomitant antibodies target different muscle proteins, including titin, myosin, tropomyosin, and the ryanodine receptor (RyR) (3–6). It is generally believed that pathogenic antibodies are closely related to the occurrence, development, and prognosis of autoimmune diseases (7–10). Although the pathogenicity of these concomitant antibodies is unclear, diagnostic and prognostic values for Titin and RyR antibodies have already been established based on intracellular localization of their target antigens (4, 11, 12). Serum antibody detection also plays an important role in the clinical diagnosis of MG, and more than one autoantibody against extracellular or intracellular targets has been noted in patients with MG (13, 14). However, the clinical value of these antibodies remains unclear. In this study, we retrospectively analyzed the prevalence, clinical features, and prognosis of anti-AChR positive MG combined with anti-LRP4 or anti-Titin antibodies.

## Methods

### Patient Information

Medical records and follow up data from 1,109 MG patients who were treated in our hospital between January 2013 and December 2019 were retrospectively reviewed and analyzed. The inclusion criteria included: (1) Patients who had been diagnosed with MG and were over 18 years of age. The MG diagnosis was based on fluctuating weakness symptoms along with supporting pharmacological, serologic, and electrophysiologic tests; (2) Onset symptoms and signs were compatible with generalized MG; (3) Patients were not treated with steroids, immunosuppressive agents, IVIG, or plasma exchange for at least 6 months before antibody detection; (4) Anti-AChR, MuSK, LRP4, and Titin-Ab were measured; (5) Patients were seropositive for anti-AChR antibody.

**Figure 1:**
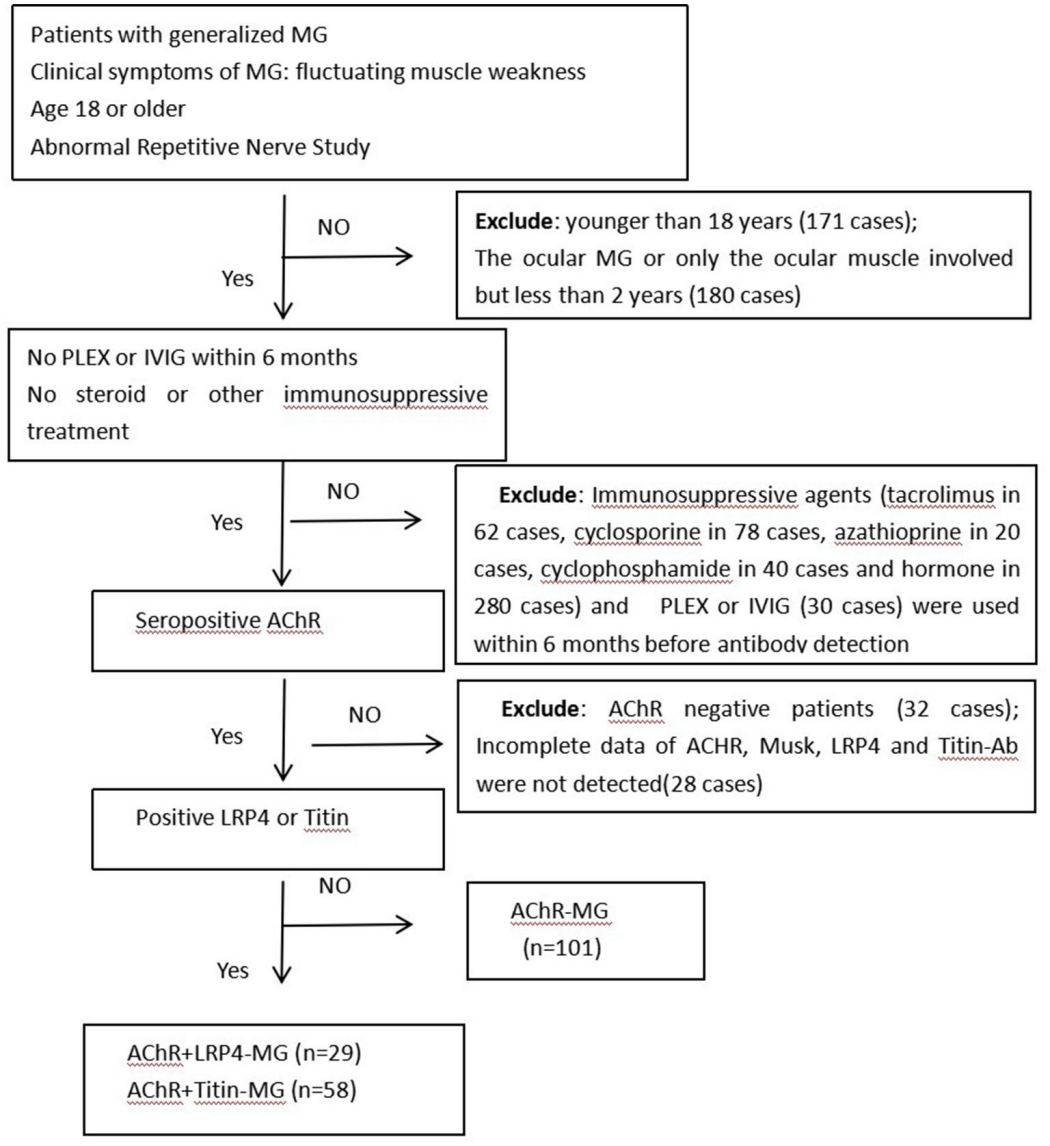
Flowchart of participants included in this study. Abbreviations: MG, Myasthenia gravis; PLEX, plasma exchange; IVIG, intravenous immunoglobulin; AChR, acetylcholine receptor; LRP4, low-density lipoprotein receptor-related protein 4; n, number of patients.

The following patients were excluded from the study: (1) A total of 171 patients who were younger than 18 years old at the time of admission; (2) Patients who had ocular MG or who only had ocular muscle involvement but for <2 years (180 cases); (3) Patients who had been treated with immunosuppressive agents (tacrolimus in 62 cases, cyclosporine in 78 cases, azathioprine in 20 cases, cyclophosphamide in 40 cases and steroids in 280 cases), had plasma exchanges (PLEX) or had intravenous immunoglobulin (IVIG) treatment (30 cases) within 6 months prior to antibody detection; (4) Patients who were negative for anti-AChR (32 cases); (5) Patients who had incomplete data regarding anti-AChR; or within whom Musk, LRP4, and Titin-Ab were not detected (28 cases). Pregnant individuals were also excluded in this study. We ultimately enrolled 188 patients in our study (Figure 1).

Myasthenia gravis was diagnosed by senior neurologists based on the guidelines of the International Consensus Guidance for Management of Myasthenia Gravis (2, 5). Patients were classified into three groups: AChR-MG, AChR+LRP4-MG, and AChR+Titin-MG. Clinical, diagnostic, therapeutic, and prognosis data, including gender, age of onset, initial symptoms, disease progression, clinical classification, disease severity, the incidence of myasthenia crisis, thymus histopathology, therapeutic options, and prognosis were collected.

### Antibody Testing

All patients were tested for MG-related antibodies in the serum before immunotherapy. If AChR Ab was positive, MuSK, LRP4, Titin, and RyR Ab were further tested in these patients.

A radioimmunoassay for the AChR antibody was performed according to the manufacturer's protocol (RSR Limited, United Kingdom). Patients were defined as antibody positive if antibody titers were ≥0.5 nmol/l (AChR Ab). Blood was tested for MuSK, LRP4, and Titin using enzyme-linked immunoassay (ELISA) as previously described (15). The investigators who performed the ELISA experiments were blinded to clinical diagnoses.

### Therapy

Therapeutic strategies for generalized MG include acetylcholinesterase inhibitors and immunotherapeutic agents. Symptomatic treatment with oral pyridostigmine bromide was used in patients who responded positively to the neostigmine trial. Most patients with generalized MG require induction therapy with glucocorticosteroids. During therapeutic periods, the steroid dosage was gradually increased or decreased according to patients' conditions. Immunosuppressive agents, including azathioprine, cyclosporine A, tacrolimus, or cyclophosphamide, were used in combination with corticosteroids if needed. If the disease was severe (i.e., involved respiratory muscle and bulbar muscle), patients were treated with IVIG and plasma exchanges. These patients were followed up for 2 years. Patients who received either azathioprine, tacrolimus, cyclosporine, or cyclophosphamide were considered to receive immunosuppressive therapy.

### Prognosis

Clinical status and disease severity were evaluated based on MGFA classifications, quantitative MG scores (QMGs), and the daily living scale (MG-ADL), respectively. In terms of MGFA post-intervention status (PIS), the classification of “Minimal Manifestation Status (MMS) or better” included minimal manifestation Status (MM0-3), pharmacological remission (PR), and complete stable remission (CSR).

All individuals were followed up and evaluated for 2 years after different treatments. The study was stopped after 2 years. The proportion of patients in the three groups who reached an "MMS or better” state after treatment, and maintained it for more than 6 months were analyzed.

### Statistical Analysis

SPSS 26.0 statistical software (IBM, Armonk, New York) was used for statistical analysis. Categorical data were represented as frequencies (%). Continuous data were represented as mean±standard deviation (SD), and ANOVA tests were used for quantitative data. The median (interquartile interval) was used for non-normally distributed statistical descriptions, and nonparametric tests were used for inter-group comparisons. Qualitative statistics were evaluated using two-tailed Fisher's exact tests. A *P* < 0.05 was considered statistically significant.

## Results

### Demographic Information

Of the 188 seropositive generalized MG patients, 29 patients were positive for both AChR and LRP4 antibodies, while 58 cases were positive for both AChR and Titin. The mean age of disease onset was 47.41 ± 7.0, 49.81 ± 9.2, and 48.11 ± 6.5 years in the AChR-MG, AChR+LRP4-MG, and AChR+Titin-MG groups, respectively. AChR+LRP4-MG showed female predominance (27.6 vs. 72.4%). The proportion of men and women in the AChR-MG and AChR+Titin-MG groups was relatively equal (48.5 vs. 51.5, 58.6 vs. 41.4%; respectively), and there were no significant gender differences between the two groups (Table 1).

**Table 1:** Characteristics of patients with AChR-MG, AChR+LRP4-MG and AChR+Titin-MG.

	**AChR MG** **(*n* = 101)**	**AChR+LRP4 MG (*n* = 29)**	**AChR+Titin MG (*n* = 58)**	***P*-value** **(AchR+LRP4 MG vs. AchR MG)**	***P*-value** **(AchR+Titin MG vs. AchR MG)**
Sex					
Men	49 (48.5%)	8 (27.6%)	34 (58.6)	0.045	0.219
Women	52 (51.5%)	21 (72.4%)	24 (41.4%)		
Onset age (years)	47.41 ± 7.0	49.81 ± 9.2	48.11 ± 6.5	0.498	0.809
Onset distribution					
Ocular	86 (85.1%)	20 (69.0%)	49 (84.5%)	0.000	0.481
Bulbar	8 (7.9%)	1 (3.4%)	7 (12.1%)		
Limb	7 (6.9%)	8 (27.6%)	2 (3.4%)		
Time from ocular onset to other muscle (months)	11.69 ± 0.0	13.08 ± 0.5	5.14 ± 0.0	0.472	0.000
Myasthenic crisis	18(17.8%)	2(6.9%)	15(25.9%)		
Thymoma	20(19.8%)	1(3.4%)	19(32.8%)	0.068	0.035
MGFA					
IIa	25 (24.8%)	14 (48.3%)	7 (12.1%)	0.02	
IIIa	4 (4.0%)	1 (3.4%)	4 (6.9%)		
IVa	2 (2.0%)	0 (0)	0 (0)		
IIb	15 (14.9%)	5 (17.2%)	4 (6.9%)		
IIIb	21 (20.8%)	2 (6.9%)	15 (25.9%)		
IVb	16 (15.8%)	5 (17.2%)	14 (24.1%)		
V	18 (17.8%)	2 (6.9%)	14 (24.1%)		
QMG scores	13 (8–19)	9 (6–14)	15.5 (11.8–22.5)	0.008	0.005
MG ADL scores	8 (5–13)	6 (4–8)	10 (8–13)	0.009	0.018
Thymectomy	26 (25.7%)	3 (10.3%)	22 (37.9%)	0.127	0.151

*Comparison of clinical data among the three groups was done by ANOVA test or Fisher exact test or nonparametric test. Abbreviations: MG, Myasthenia gravis; AChR, acetylcholine receptor; LRP4, low-density lipoprotein receptor-related protein 4; MGAF, Myasthenia Gravis Foundation of America; QMG, quantitative MG score; MG-ADL, MG-specific activities of daily living scale. The data are shown as mean±SD or ratio or median (interquartile interval)*.

### Clinical Characteristics

Ocular muscle weakness was the most common onset symptom in all three groups. More patients in the AChR+LRP4-MG suffered from limb weakness onset than in the AChR-MG and AChR+Titin-MG groups (27.6 vs. 6.9% and 11.5%; *P* < 0.001). Compared to the AChR-MG and AChR+LRP4-MG groups, patients in the AChR+Titin-MG group tended to have shorter conversion times from ocular to generalized MG (5.14 ± 0.0 vs. 11.69 ± 0.0 and 13.08 ± 0.5 months; *P* < 0.001), Furthermore, AChR+Titin-MG patients had greater bulbar dysfunction, higher incidences of thymoma (32.8 vs. 19.8 and 3.4%; *P* = 0.006), and more severe QMG scores [15.5 (11.75–22.5) vs. 13 (8–19) in AChR-the MG group (*P*=0.005), and 9 (6–14) in the AChR+LRP4-MG group (*P* < 0.001)]. MG-ADL scores were also significantly increased in the AChR+Titin-MG group [10 (8–13) vs. 8 (5–13) in the AChR-MG group, *P* = 0.018; and 6 (4–8) in the AChR+LRP4-MG group, *P*< 0.001].

The most common MGFA classification in AChR+LRP4-MG patients was MGFA IIa (48.3%), while 25.9% of AChR+Titin-MG patients were classified as MGFA IIIb. Additionally, more patients were classified as MGFA IVb-V in the AChR+Titin-MG group than in either the AChR-MG and AChR+LRP4-MG groups (48.2 vs. 33.2, and 24.1%, *P* = 0.02).

**Figure 2:**
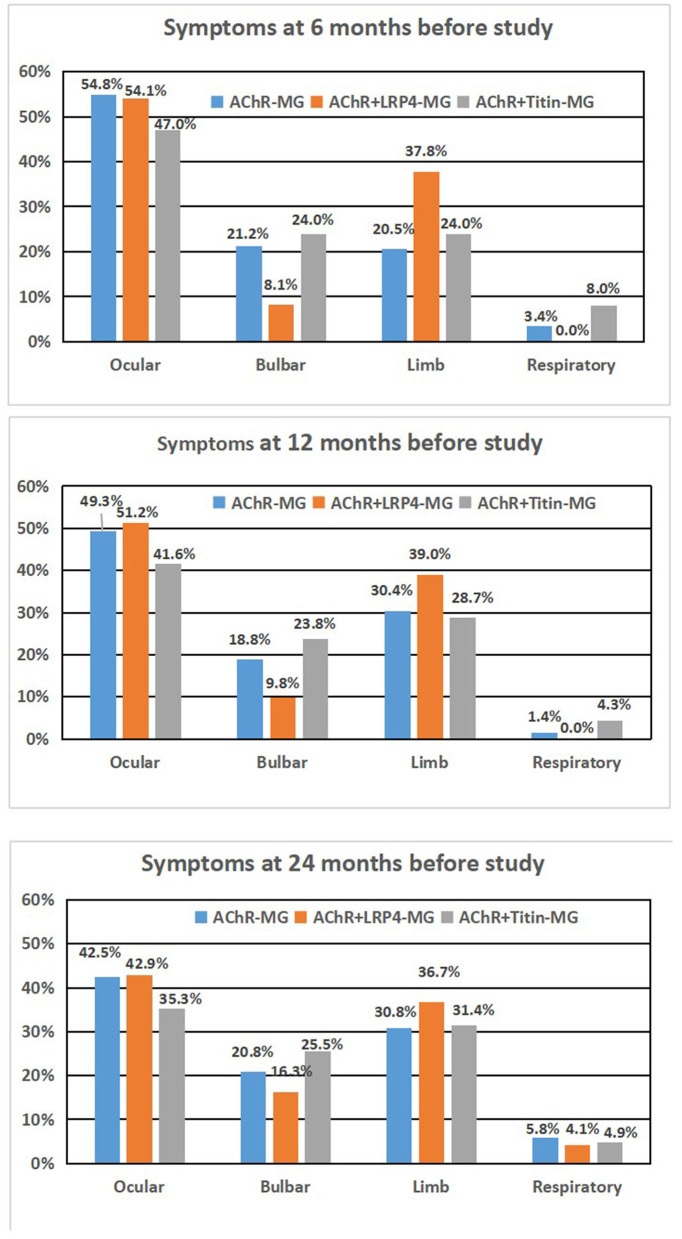
Syptoms of AChR-MG, AChR+LRP4-MG, and AChR+Titan-MG patients during different time period. **(A)** 6 months before study, **(B)** 12 months before study, and **(C)** 24 months before study.

Affected muscles in the three groups were analyzed at different time points (6 months, 12 months, and 24 months; see Figure 2). Our results showed that clinical symptoms did not differ significantly among the three groups during different time points.

### Treatment and Prognosis

Patients were treated with standard therapies for MG. The rates of achieving MMS or better in the three groups within 2 years after immunosuppressive treatment were 51.5, 62.1, and 51.7%, respectively (Table 2). AChR+Titin-MG treatment was highly dependent on steroids combined with immunosuppressive agents (Table 3).

**Table 2:** Comparison of the prognosis after immunosuppressive therapy among AchR-MG, AchR+LRP4 and AchR+Titin MG.

	**AchR-MG**	**AchR+LRP4**	**AchR+Titin**	***P*-value**
MM	52/101 (51.5%)	18/29 (62.1)	30/58 (51.7%)	0.581
MM-0 or better	9/101 (8.9%)	2/29 (6.9%)	2/58 (3.4%)	0.454
MM-1	10/101 (9.9%)	5/29 (17.2%)	4/58 (6.9%)	0.358
MM-2	15/101 (14.9%)	6/29 (20.7%)	8/58 (13.8%)	0.799
MM-3	18/101 (17.8%)	5/29 (17.2%)	16/58 (27.6%)	0.31

*Minimal Manifestations (MM), The patient has no symptoms of functional limitations from MG but has some weakness on examination of some muscles. This class recognizes that some patients who otherwise meet the definition of CSR or PR do have weakness that is only detectable by careful examination; MM-0, The patient has received no MG treatment for at least 1 year; MM-1, The patient continues to receive some form of immunosuppression but no cholinesterase inhibitors or other symptomatic therapy; MM-2, The patient has received only low-dose cholinesterase inhibitors (<120 mg pyridostigmine/day) for at least 1 year; MM-3, The patient has received cholinesterase inhibitors or other symptomatic therapy and some form of immunosuppression during the past year*.

**Table 3:** Therapeutic strategy for MMS or better among three groups.

	**AchR MG**	**AchR+LRP4** **MG**	**AchR+Titin** **MG**	***P*-value**
steroid	14 (26.9%)	5 (27.8%)	6 (20.0%)	0.819
Immunosuppressant	28 (53.9%)	10 (55.6%)	7 (23.3%)	0.017
steroid+ Immunosuppressant	10 (19.2%)	3 (16.7%)	17 (56.7%)	0.001

## Discussion

Our study found that clinical symptoms of anti-AChR MG combined with anti-LRP4 or anti-Titin antibody were more severe and progressed faster than anti-AChR positive MG. Regardless of antibody status, all patients responded well to immunotherapy and had relatively good prognoses.

### Demographic Characteristics

About 85% of MG patients have autoantibodies against AChR, whereas 5%-26% of MG have autoantibodies against MuSK (1–3, 15–17). We focused on AChR positive patients in this study. We only found one patient who was both AChR and MuSK antibody-positive, which was lower than the proportion of patients who had seronegative MG (and who were not excluded in our study).

Most previous studies on LRP4 have largely focused on the seronegative MG population. However, there have been some reports of anti-AChR patients with double-positive LRP4 antibodies in clinical practice (20). Among 1,109 patients diagnosed with MG at our center, we found 29 cases with AChR combined with anti-LRP4 antibodies. The proportion of patients with anti-LRP4-antibodies was 2.61%, which coincided with the proportion in double-negative patients (14) and in other studies (18–20). Titin auto-antibodies were found in 30.9% of seropositive patients [compared with the 20–40% that has previously been reported in the literature (11, 13, 21). We found that the AChR+LRP4-MG phenotype showed a strong female predominance (72.4%), which was consistent with previous studies (22, 23). The mean age of onset in the three groups was 47.41 ± 7.0, 49.81 ± 9.2, and 48.11 ± 6.5 years, respectively. We focused on adults with generalized MG and excluded relatively young ocular patients.

### Clinical Features

Most MG patients with ocular symptoms at onset may progress to the generalized form of the disease within 2 years (1, 3). Our results confirmed that most MG patients had an ocular-only onset. However, the AChR+LRP4-MG group had significantly higher numbers of patients with limb weakness during disease onset than the AChR-MG or AChR+Titin-MG group. Therefore, AChR+LRP4-MG patients were much more likely to have generalized MG at the time of disease onset. Three of the AChR+LRP4-MG patients presented with MGFA class V in our study (20). It is unknown if other antibodies, such as agrin, were positive because that testing was not done (14).

Compared to the AChR-MG and AChR+LRP4-MG groups, AChR+Titin-MG patients showed shorter progression times from ocular to generalized MG (within 5.1 months). Rapid disease progression following symptom onset maybe because of the involvement of titin antibodies. Additionally, our data on MGFA classifications showed that 25.9% of AChR+Titin-MG patients were classified as MGFA IIIb, while 48.3% of AChR+LRP4-MG patients were classified as MGFA IIa. Moreover, there were more patients with MGFA IVb -V. Our results indicated that AChR+Titin-MG was associated with more severe disease status. Titin antibodies are usually considered to be accompanying antibodies and can only be found in patients with MG and anti-AChR antibodies. It is highly likely that the presence of thymoma in AChR+Titin-MG patients is related to their disease pathology.

Muscles that were involved at different time points (i.e., 6, 12, and 24 months before our study) did not differ significantly among the three groups. Thus, affected muscle groups appear to be similar at different stages of the disease, although disease severity differs.

### Treatment and Prognosis

Current common treatments for MG include AChE inhibitors, immunosuppressive drugs, thymectomy, IVIG, and plasmapheresis (24, 25). In our study, the proportions of patients who have achieved MM-3 or better for more than 6 months in the three groups were 51.5, 62.1, and 51.7%, respectively. These percentages are higher than what was reported in a study conducted by Utsugisawa K (26) but are consistent with other previous studies (27, 28).

All patients were treated with pyridostigmine. Monotherapy with an immunosuppressive agent was used in 53.9 and 55.6% of AChR-MG and AChR+LRP4-MG patients, and immunosuppressive therapy was used in combination therapy with azathioprine or tacrolimus corticosteroids in 56.7% of patients with AChR+Titin-MG.

The proportion of steroids combined with immunosuppressive agents in the AChR+Titin MG group was much higher than in the other two groups, suggesting that AChR+Titin MG needs stronger immunotherapy to achieve the same outcomes and is thus also associated with severe immune dysfunction.

In summary, anti-AChR positive MG can coexist with anti-LRP4 or anti-Titin antibodies. AChR+LRP4-MG has a female predominance and presents with milder symptoms. Furthermore, AChR+Titin-MG shows a shorter conversion time from ocular to generalized MG, a higher incidence of thymoma, and has a more severe presentation than AChR+LRP4-MG. Regardless of antibody status, all patients responded well to immunotherapy.

## Data Availability Statement

The original contributions presented in the study are included in the article/supplementary material, further inquiries can be directed to the corresponding author/s.

## Ethics Statement

The studies involving human participants were reviewed and approved by The First Medical Center of PLA General Hospital. The patients/participants provided their written informed consent to participate in this study. Written informed consent was obtained from the individual(s) for the publication of any potentially identifiable images or data included in this article.

## Author Contributions

YC and XT acquired the clinical data, reviewed the literature, and drafted the article. JH and FQ designed the study, supervised the initial drafting, and critically revised the article. YW, SX and YY collected and analyzed the clinical data. All authors contributed to the article and approved the submitted version.

## Conflict of Interest

The authors declare that the research was conducted in the absence of any commercial or financial relationships that could be construed as a potential conflict of interest.

## Publisher's Note

All claims expressed in this article are solely those of the authors and do not necessarily represent those of their affiliated organizations, or those of the publisher, the editors and the reviewers. Any product that may be evaluated in this article, or claim that may be made by its manufacturer, is not guaranteed or endorsed by the publisher.
